# Prolonged milk provisioning and extended maternal care in the milking spider Toxeus magnus: biological implications and questions unresolved

**DOI:** 10.24272/j.issn.2095-8137.2019.041

**Published:** 2019-07-18

**Authors:** Bing Dong, Rui-Chang Quan, Zhan-Qi Chen

**Affiliations:** 1 CAS Key Laboratory of Tropical Forest Ecology, Xishuangbanna Tropical Botanical Garden, Chinese Academy of Sciences, Xishuangbanna Yunnan 666303, China; 2 Center for Integrative Conservation, Xishuangbanna Tropical Botanical Garden, Chinese Academy of Sciences, Xishuangbanna Yunnan 666303, China; 3 University of Chinese Academy of Sciences, Beijing 100049, China

**Keywords:** *Toxeus magnus*, Spider milk, Parental care, Food provisioning, Lactation

## Abstract

Prolonged milk provisioning and extended parental care for nutritionally independent offspring, previously considered to only co-occur in long-lived mammals (Clutton-Brock, 1991; Royle et al., 2012), were recently reported in the reproduction of the milking spider, *Toxeus magnus* (Chen et al. 2018). Newly hatched *T. magnus* spiderlings require 53 days to develop to maturity, with an average adult body length of 6.6 mm. The mother provides milk droplets to her newly hatched spiderlings until they develop into subadults (~38 days old), during which their body lengths increase from 0.9 mm at birth to 5.3 mm at weaning. Although spiderlings can forage for themselves at around 20 days old, they remain in the breeding nest for weeks after maturity.

*T. magnus* is not the first recorded invertebrate to provide a milk-like substance to nourish offspring. Examples of maternal nutrient liquid provisioning are reported across different arthropod clades, such as flies, cockroaches, burying beetles, bees, swaps, and ants (Benoit et al., 2015; Bilinski et al., 2018; Ostrovsky et al., 2016; Wong et al., 2013). Examples also appear in Arachnida, including scorpions and pseudoscorpions (Ostrovsky et al., 2016). However, there are two distinct differences that emphasize the uniqueness of *T. magnus* compared with the above examples. First, *T. magnus* mothers progressively feed their young with secretions from the hatchling stage until sub-adulthood, whereas those mentioned above only provide nutritional liquid pre-birth, known as insect viviparity, or for newly-hatched young. Second, *T. magnus* provides maternal care for sexually mature offspring, which has not been reported in other examples. These two characteristics suggest that *T. magnus* is comparable to long-lived social mammals in reproduction.

Milk is a unique type of maternally secreted fluid found across the animal kingdom and plays an integrative role in nourishment and immunological protection for young (Ward & German, 2004). Nutritionally, essential and non-essential amino acids and bioactive proteins in milk are indispensable for newborn survival (Demmelmair et al., 2017). In addition to nourishment and immunological functions, milk provisioning in mammals can further affect litter size, sex ratio, offspring size, and brain size at weaning (McCGraham, 1964; McClure, 1987; Page et al., 2009; Taylor et al., 2005). However, despite the unique benefits, lactation is considered the costliest part of reproduction for mothers (Gittleman & Thompson, 1988), with a mean caloric intake for females during lactation 66%–188% greater than that for non-reproductive females (Gélin et al., 2015; Glazier, 1985; McCGraham, 1964; Millar, 1978). In addition to the costs of lactation, the deposition of fat prior to breeding and during gestation may function as a supplementary energy supply to meet the needs of milk production (George et al., 2010; McClure, 1987). We speculate that the extremely high cost of milk provisioning is one of the main barriers for the evolution of lactation in non-mammals. The discovery of similar milking behavior of *T. magnus* implies that tiny-bodied arthropods can afford the costs of “lactation”; however, what costs are incurred by mothers in regard to this “lactation” remain unidentified. Therefore, prospective studies are required to examine the costs of milk provisioning to the mother, as well as future reproduction opportunities, longevity, predation pressure, and energy costs of milk production. These investigations should help clarify the specific factors that contribute to the occurrence of “lactation”.

Mammalian milk is secreted from the mammary gland, which is considered to have evolved from sweat or skin glands, with the nipple thought to have evolved from relevant hair follicles (Clutton-Brock, 1991; Royle et al., 2012; Vorbach et al., 2006). There are three possible criteria for the origin of the mammary gland: (1) enhancement of offspring survivorship at the early birth stage by supplying additional nutrition (Hayssen, 1993), (2) provision of water to keep the early mammalian parchment-like eggs moist (Oftedal, 2002), and (3) establishment of immunological protection for young to reduce infection risks (Vorbach et al., 2006). In *T. magnus*, however, we found no evidence for the physiological process of milk secretion or immunological effects of milk. Hence, histological, transcriptomic, and metabolic analyses are required to confirm where and how the spider milk is secreted. Furthermore, behavioral and biochemical studies are necessary to examine the immunological functions of milk. These results should enhance our understanding on the origin and evolution of milk secretions across different animal clades.

Parental care often stops once offspring acquire the ability to forage for themselves (Royle et al., 2012). Caring for nutritionally independent offspring is unusual, with exceptions mainly reported in long-lived vertebrates, such as the bonobo (*Pan paniscus*) (Clutton-Brock, 1991; Royle et al., 2012). The rarest form of parental care is for sexually mature offspring, which appears to be restricted to long-lived social vertebrates (Clutton-Brock, 1991; Royle et al., 2012). This prolonged parental care may increase fitness by enabling offspring to devote more time to learning life skills, such as foraging (Hoppitt et al., 2008), anti-predation, defense against brood parasites, social skills, and mate selection (Brown & Laland, 2001; Curio, 1993; Davies & Welbergen, 2009). For example, bonobo mothers usually assistant their sexually mature sons to win intrasexual competitions for social status and mates (Surbeck et al., 2011). The milking spider, *T. magnus*, is the first recorded invertebrate species to provide parental care to adult offspring, although it remains unknown whether offspring gain long-term benefits from extended parental care as obtained in long-lived social vertebrates. Therefore, further research is required to examine the correlations between extended parental care and long-term fitness of offspring. Future studies should examine whether offspring provided with extended parental care are more competitive than those without when competing for high-quality mates and food resources. Furthermore, if extended parental care enhances offspring fitness, how are the long-term benefits achieved? Such studies will greatly expand our knowledge on the long-term benefits of extended parental care in a wider range of animal clades.

The evolutionary process and current “lactation” in the spider clade (Araneae) are also worth investigating, which will help to understand the evolutionary history and current patterns and factors that contribute to or restrict milk provisioning and extended parental care in non-mammals. Thus, additional species from the same family (Salticidae) and across other families are required for molecular and behavioral phylogenic reconstruction. Furthermore, genomic analyses of milk secretions and extended parental care in spiders remain to be undertaken.

In conclusion, *T. magnus* is the first non-mammalian species found to provide both milk and extended maternal care for their young (Chen et al., 2018), which was previously thought to only co-occur in long-lived social vertebrates. Based on the current results, a series of focused studies, ranging from ecological to genetic research, should be conducted to further our understanding of the evolution and adaptation of lactation and extended parental care in non-mammals.
